# Otitis media in children vaccinated during consecutive 7-valent or 10-valent pneumococcal conjugate vaccination schedules

**DOI:** 10.1186/1471-2431-14-200

**Published:** 2014-08-11

**Authors:** Amanda Jane Leach, Christine Wigger, Ross Andrews, Mark Chatfield, Heidi Smith-Vaughan, Peter Stanley Morris

**Affiliations:** 1 Menzies School of Health Research, Charles Darwin University, Darwin, NT, Australia; 2 Royal Darwin Hospital, Darwin, NT, Australia

**Keywords:** Otitis media, Child, Indigenous, Pneumococcal vaccines, Prevalence, Public health, Surveillance, Risk factors

## Abstract

**Background:**

In 2001 when 7-valent pneumococcal conjugate vaccine (PCV7) was introduced, almost all (90%) young Australian Indigenous children living in remote communities had some form of otitis media (OM), including 24% with tympanic membrane perforation (TMP). In late 2009, the Northern Territory childhood vaccination schedule replaced PCV7 with 10-valent pneumococcal *Haemophilus influenzae* protein D conjugate vaccine (PHiD-CV10).

**Methods:**

We conducted regular surveillance of all forms of OM in children in remote Indigenous communities between September 2008 and December 2012. This analysis compares children less than 36 months of age who received a primary course of at least two doses of PCV7 or PHiD-CV10, and not more than one dose of another pneumococcal vaccine.

**Results:**

Mean ages of 444 PCV7- and 451 PHiD-CV10-vaccinated children were 20 and 18 months, respectively. Bilaterally normal middle ears were detected in 7% and 9% respectively. OM with effusion was diagnosed in 41% and 51% (Risk Difference 10% [95% Confidence Interval 3 to 17] p = 0.002), any suppurative OM (acute OM or any TMP) in 51% versus 39% (RD −12% [95% CI −19 to −5] p = 0.0004], and TMP in 17% versus 14% (RD −3% [95% CI −8 to 2] p = 0.2), respectively. Multivariate analyses described a similar independent negative association between suppurative OM and PHiD-CV10 compared to PCV7 (Odds Ratio = 0.6 [95% CI 0.4 to 0.8] p = 0.001). Additional children in the household were a risk factor for OM (OR = 2.4 [95% CI 2 to 4] p = 0.001 for the third additional child), and older age and male gender were associated with less disease. Other measured risk factors were non-significant. Similar clinical results were found for children who had received non-mixed PCV schedules.

**Conclusions:**

Otitis media remains a significant health and social issue for Australian Indigenous children despite PCV vaccination. Around 90% of young children have some form of OM. Children vaccinated in with PHiD-CV10 had less suppurative OM than children vaccinated with PCV7. Ongoing surveillance during the PCV13 era, and trials of early intervention including earlier and mixed vaccine schedules are warranted.

## Background

Community based surveillance pre- and post- introduction of seven-valent pneumococcal conjugate vaccine (PCV7) indicates that less than 10% of Australian Indigenous children living in remote Northern Territory (NT) communities have normal middle ears and around 20% have tympanic membrane perforation, TMP (either acute otitis media with perforation (AOMwiP), dry perforation (DP), or chronic suppurative otitis media (CSOM)) [1,2]. *Streptococcus pneumoniae* (pneumococcus) and non-typeable *H. influenzae* (NTHi) are major pathogens detected by culture [2,3] or PCR [4] in ear discharge of children with AOMwiP or CSOM. PCV7 has greatly reduced vaccine-serotype invasive pneumococcal disease (IPD) and has indirect protective effects via reduced carriage [5]. Replacement by non-vaccine serotypes [6] may limit the extent and persistence of this benefit [7]. In addition to protection from three additional serotypes, clinical trial data suggested that the 10-valent pneumococcal *Haemophilus influenzae* protein D conjugate vaccine (PHiD-CV10) may protect against NTHi otitis media [8], although Australian regulatory authorities have not approved a license indication for the latter [9]. The NT infant vaccination schedule changed from a combination PCV7/PPV23 to a 3 + 1 PHiD-CV10 schedule (Table 1). Through ongoing surveillance of otitis media over this period, we were able to compare the community level impact of PCV schedule on OM and explore potential associations between OM severity and risk factors. Our primary hypotheses were that in children 6 months to 36 months of age the prevalence of TMP (AOMwiP, DP and CSOM) would be less, and the prevalence of bilateral normal middle ears would be higher in PHiD-CV10 vaccinated children compared to PCV7 vaccinated children.

**Table 1:** Pneumococcal vaccines in the childhood vaccination schedule for Northern Territory Indigenous children

**Date commenced**	**Vaccine**	**Age (mo)**
1^st^ July 2001	PCV7 & PPV23	2,4,6 18
1^st^ October 2009*	PHiD-CV10	2,4,6,18
1^st^ October 2011	PCV13	2,4,6,18

*No other Australian jurisdiction recommended that PHiD-CV10 replace PCV7.

## Methods

### Study design, setting, community recruitment and ethical approval

Since 2001 a total of thirty five remote communities in the NT [6] and one in Western Australia have participated in at least one community-based cross sectional survey of otitis media and nasopharyngeal carriage. This report includes cross sectional data from 25 communities participating between September 2008 and December 2012. The study was approved by the Human Research Ethics Committee of the Northern Territory Department of Health and the Menzies School of Health Research (EC00153), and the Western Australian Aboriginal Health Information and Ethics Committee (WAAHIEC). Each community council provided written approval of the study to the Ethics committee. Our research has adhered to the STROBE guidelines as outlined at http://www.strobe-statement.org.

### Participant recruitment and consent

In these remote communities where the birth cohort is between 5 and 45 infants per year, we aimed primarily to see all children under 36 months of age as well as older children (up to 6 years of age) if available. No random selection methods were used. Individual families were approached with information about the study. Consent was sought from parents for their child (regardless of ear health status or history) to have an ear examination, nasal swab, swab of ear discharge if present, and general child health check. Parents or carers were also asked for permission to access the mother’s and the child’s medical records and complete a lifestyle interview regarding information on likely risk or protective factors for otitis media.

### Inclusion and exclusion criteria

Aboriginal children between 0 and 6 years of age, resident in participating communities, and whose parents or carers provided signed consent, were eligible for surveillance. For this report we limit analysis as described below (Statistical analysis).

### Clinical assessments

#### Ear examinations and general health assessments

All clinical assessments were made by ear health research nurses with extensive training in the diagnosis and management of otitis media in this population. Otoscopic findings were recorded on a standardized form. Assessments were made using a tympanometer (Grason Stadler GSI 38), a LumiView (Welch Allyn) with Siegel’s speculum for pneumatic otoscopy, and a video-otoscope (Welch Allyn macroview or MedRx video-otoscopes).

#### General health measures

Common conditions of childhood were recorded at the time of ear assessment by direct observation; the child’s skin (head, arms, legs and trunk) was examined for the presence of scabies, tinea, skin sores or other skin condition; presence of nasal discharge (visible at a distance of 1 meter), and any cough (spontaneous or cough on request, either wet or dry). We categorised ‘not sure’ as absent. Antibiotics and other treatments or referrals were provided to participants according to local guidelines.

#### Definitions of OM

We categorized middle ear states as follows: (1) normal; (2) otitis media with effusion (OME); (3) acute otitis media without perforation (AOMwoP); (4) AOM with perforation (AOMwiP); (5) dry perforation; and (6) chronic suppurative otitis media (CSOM). The final middle ear diagnosis reflected the child’s more severely affected ear (highest category). We based our criteria for diagnosis on recommendations for clinical practice in this population [10]: (i) OME – intact and non-bulging tympanic membrane (TM) and Type B tympanogram; (ii) AOMwoP – any bulging of the TM and Type B tympanogram; (iii) AOMwiP – middle ear discharge observed and TM perforation recently healed or present for less than six weeks or covering less than 2% of the pars tensa of the TM; (iv) dry perforation – TM perforation without any discharge observed; (v) CSOM – middle ear discharge observed and perforation present for longer than six weeks and covering at least 2% of the pars tensa of the TM. We also include combination categories of any suppurative OM (any AOM, AOMwiP or CSOM) and any TMP (any AOMwiP, dry perforation or CSOM). Where duration of discharge was not known, size of perforation was used to distinguish AOMwiP and CSOM. Where otosocopy was not successful we used the child’s tympanometry result and defined the child’s status as OME if either ear had a Type B tympanogram. We asked the mother if she thought her child had ear pain that day or during the previous evening. These a priori diagnostic criteria have been applied in all our surveillance and clinical trials conducted in this population since 2001 [1].

#### Medical record review

The child’s medical records were reviewed to obtain dates of vaccinations, recent clinic presentations, antibiotics prescribed within the previous 5 weeks, sex, gestational age, date of birth, birth weight, and latest haemoglobin result.

### Risk factor questionnaires

The parent or guardian (usually the mother) was asked a standardised set of questions about common risk factors for OM, including the number of siblings, numbers of people and children (less than 5 years of age) living in the child’s house, whether the mother or child’s siblings had ever had TMP (“runny ears”), her highest level of education, if she smoked, and whether the child was exposed to campfire smoke within the previous week, the number of days per week that the child attended child care, whether the child washed with soap the previous day, had ever used a pacifier, or was ever breastfed.

### Statistical analysis

We compared children who had received at least 2 doses of PCV7 with children who had received at least 2 doses of PHiD-CV10 as their primary course vaccines, with or without a subsequent single dose of an alternative PCV, or PPV23. For this analysis we included children 0 to 36 months of age.

Confidence intervals (CI, 95%) and risk differences (RD, 95% CI) were calculated where appropriate. Stata version 12 was used for all data analyses [11].

Multivariate logistic regression adjusting for community was undertaken to examine risk factors, including vaccine type, for our combination category of any suppurative OM. For comparisons of children with and without any suppurative OM, children with dry perforation were excluded. A global test was performed for categorical risk factors.

#### Missing data

If participants declined a clinical assessment or a swab, the data were coded as missing. If parents or carers refused or were unsure of their response to an interview question, we also coded this as missing. Missing data were then excluded from the denominator for summary statistics.

## Results

### Participant exclusions and PCV vaccination status

We enrolled 1,027 children and made 1,088 child visits. Only the first visit per child is included in this analysis. Forty one children had received PCV13 only. We excluded a further 91 children on the basis of their vaccination status; 33 children who had not been vaccinated, 25 who had only a single dose of one or both vaccines, 30 children who had received 2 doses of each vaccine, two children who received two doses of PCV13, and one child who had received two or more doses of more than one PCV. Of 895 children in this analysis, 444 were in the PCV7 group and 451 in the PHiD-CV10 group. All had received two or more doses of appropriate vaccine and not more than one dose of any other PCV. Most children had received 3 or more PCV doses (Table 2).

**Table 2:** Number of doses of pneumococcal vaccine received by 444 PCV7 children, 451 PHiD-CV10 children

**Doses of PCV7**	**Doses of PHiD-CV10**					
	**0**	**1**	**2**	**3**	**4**	**TOTAL**
0	33^e^	18 ^e^	50	284	77	411
1	5 ^e^	2 ^e^	23	16	1	40
2	28	15	30 ^e^			43
3	322	73	1 ^e^			395^¥^
4	5	1				6
+ PPV23^§^	138^§^					
+ PCV13^¥^	2^¥^	0	19^¥^	70^¥^	1^¥^	92^¥^
**TOTAL**	355	89	73	300	78	895

PCV7, 7-valent pneumococcal conjugate vaccine.

PHiD-CV10, 10-valent pneumococcal *Haemophilus influenzae* protein D conjugate vaccine.

PCV13, 13-valent pneumococcal conjugate vaccine.

Included mixed schedules:

^§^138 children also received one dose PPV23: (ceased in October 2010) 138 of 177 PCV7 3-dose children who were age-eligible for PPV23.

^¥^92 children also received one dose PCV13:

2 of 322 PCV7 3-dose children.

19 of 50 PHiD-CV10 2-dose children.

70 of 284 PHiD-CV10 3-dose children.

1 of 77 PHiD-CV10 4-dose child.

Excluded schedules

^e^91 children were excluded, including two additional children (not shown in Table 2) who received two doses of PHiD-CV10 and two doses of PCV13.

### Region and communities

All communities were in the tropical Top End region of Australia. Visits were made each year between early February and mid-December, the majority being the between April and December, during the dry and pre-cyclone season. Mobility is high amongst these families. We have previously found that around 84% of young children (<30 months of age) may be present in a community at any one visit [1]. Many additional factors influenced coverage during the course of this surveillance including Traditional Owner approvals to access the community, unscheduled cultural ceremonies such as funerals, parental consents, weather events, availability of accommodation for research staff, and the cost of travel. The sampling strategy thus resulted in differing proportions of children from 25 communities and 7 regions being seen over time (up to 44% age-eligible children were seen each year in the largest communities). Ten communities contributed at least 10 children to each of the PCV7 and PHiD-CV10 groups; 5 of these, the largest in each of 5 regions, contributed 59% data in the PCV7 group and 59% in the PHiD-CV10 group.

### Participant characteristics

#### Age, gestational age, birth weight and gender

The mean ages were 20 mo and 18 mo for PCV7 and PHiD-CV10 groups, respectively (Difference 2 months [95% CI 1 to 3] p = 0.0001). Mean gestational age and mean birth weight were significantly higher for PHiD-CV10 group. There was no significant difference in gender (Table 3).

**Table 3:** Participant characteristics, general health and antibiotics prescribed, by vaccination group

	**PCV7**		**PHiD-CV1**		**Absolute difference in mean or % [95% CI]**	P*
	**N**	**Mean (SD) or%**	**N**	**Mean (SD) or%**		
Age (mo)	444	20 (8)	451	18 (7)	2 [1-3]	0.0001
0 to <3	0		0			
3 to <6	8	2%	9	2%		
6 to <9	48	11%	60	13%		
9 to <12	39	9%	46	10%		
12 to <18	87	20%	118	26%		
18 to <24	102	23%	106	24%		
24 to <36	160	36%	112	25%		
Gestational age (weeks)	378	37.7 (2.8)	426	38.1 (2.3)	0.4 [0 to 0.7]	0.04
Birth weight (kg)	431	2.97 (0.65)	431	3.06 (0.61)	0.09 [0.00 to 0.17]	0.04
Sex (female)	210	47%	230	51%	4% [−3 to 10]	0.27
**General health**						
SKIN assessed	434/444	98%	435/451	96%		
Normal	326	75%	303	70%	−5% [−11 to 0]	0.07
Scabies	19	4%	53	12%	8% [−4 to 11]	**<0.0001**
Impetigo	62	14%	67	15%	1% [−4 to 6]	0.64
Tinea	19	2%	13	3%	1% [−1 to 3]	0.39
other	20	6%	27	6%	0% [−3 to 3]	0.96
Runny Nose	165/432	38%	198/445	44%	6% [0 to 13]	0.06
Any cough	86/436	20%	169/457	38%	18% [12 to 24]	**<0.0001**
**Other**						
Any antibiotics prescribed in previous 5 weeks	130/444	29%	189/451	42%	12% [6 to 19]	**0.0001**
Beta-lactam	101/444	23%	158	35%	12% [6-18]	**0.0001**
Macrolide	14/444	3%	18	4%	1% [−2 to 3]	0.50
Topical	18/426	4%	18/433	4%	0% [−3 to 3]	0.97
Haemoglobin (<11 mg/dL)	122/402	30%	139/403	34%	4% [−2 to 11]	0.21

*Bold signifies P value less than 0.05.

#### General health

Many children had health problems in addition to their ear disease. Overall, at least 25% children had a skin problem, mainly impetigo, round 40% had visible runny nose, and at least 20% children were diagnosed with cough. Abnormal haemoglobin (<11 mg/dL) was recorded for at least 30% children tested. Significantly more children in the PHiD-CV10 group had scabies, runny nose, or cough (12%, 44% and 38%, respectively), compared to the PCV7 group (4%, 38% and 20%, respectively). Antibiotics, predominantly beta-lactams, had been prescribed within 5 weeks of clinical assessment for 29% PCV7 children and 42% PHiD-CV10 children (RD 12% [95% CI 6 to 19] p = 0.0001) (Table 3).

#### Risk factor questionnaire respondents

Of the 444 children in the PCV7 group, 413 (93%) parents or carers consented to a structured risk factor questionnaire conducted by interview, whereas 296 (66%) parents or carers of the 451 children in the PHiD-CV10 group consented to the interview. Almost all interviewees (~97% in both groups) were the child’s mother. The mean age, gender and prevalence of any suppurative OM for these subgroups (Table 4) closely represent the whole cohort. Importantly also, the prevalence of any suppurative OM was not significantly different among PHiD-CV10 children whose parents consented to an interview and those who did not (36% versus 44% RD −8% [95% CI −17 to 2] p = 0.13, data not shown).

**Table 4:** OM risk factors, by vaccination group

	**PCV7**		**PHiD-CV10**		**Absolute difference in mean or % [95% CI]**	P*
	**N**	**Mean (SD) or %**	**N**	**Mean (SD) or %**		
**Risk factor questionnaire response**						
No parents consented	**413/444**	93%	**296/451**	66%	−27% [−32 to −22]	**<0.0001**
Mother interviewed	400/413	97%	290/296	98%	1% [−1 to 3]	0.36
**Characteristics of children in consented subgroups**						
	**PCV7**		**PHiD-CV10**			
Age (months)	413	20 (8)	296	18(7)	2.0 [0.9 to 3.2]	**0.0008**
Sex (female)	198	48%	151	51%	3% [−4 to 11]	0.42
Any Suppurative OM	208/395	53%	104/285	36%	−16% [−24 to −9]	**<0.0001**
**Risk factors**						
Crowding (persons per household)	402	8.6 (4.5)	290	8.2 (3.9)	−0.4 [−1.0 to 0.3]	0.23
Crowding (children < 5 years of age per household)	408	2.4 (1.6)	291	2.1 (1.2)	−0.3 [−0.5 to −0.1]	**0.01**
Crowding Index (%households with > 2 additional children < 5 years of age)	132/408	32%	75/291	26%	−6% [−13 to 0.2]	0.06
Child Care (any attendance > 3 days per week)	51/409	12%	41/295	14%	1.4% [−4 to 7]	0.58
Child Care (days/week)	408	0.68 (1.7)	295	0.77 (1.7)	0.09 [−0.34 to 0.16]	0.49
Washed with soap yesterday	327/362	90%	258/293	88%	−2% [−7 to 3]	0.35
Any sibling with history of “runny ears”	101/370	27%	80/249	32%	5% [−3 to 12]	0.20
Child near campfire last week	134/402	34%	88/287	31%	−3% [−10 to 4]	0.46
Maternal smoking	233/410	57%	178/283	61%	4% [−3 to 11]	0.29
Maternal age at birth of this child	297	25 (5.4)	244	26 (6.4)	1 [0.1 to 2]	**0.04**
Maternal education (certificate)	105/382	27%	60/247	24%	−3% [−10 to 4]	0.37
Never breast fed	28/409	7%	14/296	5%	−2% [−6 to 1]	0.23
Pacifier (ever)	78/404	19%	52/296	18%	−2% [−8 to 4]	0.56

*Bold signifies P value less than 0.05.

### Comparison of risk factor prevalence among PCV7 and PHiD-CV10 subgroups

Total household occupancy was not significantly different between the PCV groups, however the mean number of children less than 5 years of age per household was significantly lower in the PHiD-CV10 group (Difference −0.3 children [95% CI −0.5 to 0] p = 0.01). Other measures of household crowding, and child care attendance were not significantly different between PCV7 and PHiD-CV10 groups. For both vaccine groups, almost all children (~90%) washed with soap the previous day, around 30% children had a sibling with a history of CSOM (‘runny ears’), one third of children spent time around the campfire, and most mothers (~60%) smoked cigarettes. Mean maternal age was ~25 years, one in 5 mothers completed year 12 and one in four had a short course certificate as their highest level of formal education. Very few babies were never breast fed (~6%) and pacifier use was relatively uncommon (~20%) (Table 4).

### Primary outcome: otitis media, by vaccine group and age

#### Otitis media

Ninety seven per cent of children in each group had at least one ear successfully assessed. Four children, all in the PCV7 group, had a diagnosis based on tympanometry alone. In both groups, less than 10% had bilateral normal middle ears. However, of the ~90% with some form of OM there were vaccine group differences in OM severity. A diagnosis of OME was made for 41% PCV7 children compared to 51% PHiD-CV10 children (RD 10% [95% CI 3 to 17] p = 0.003). AOMwoP was diagnosed for 35% PCV7 children and 25% PHiD-CV10 children (RD −9% [95% CI −15 to −3] p = 0.003). Rates of AOMwiP were similar, 6% and 5% respectively (RD −1% [95% CI −4 to 2] p = 0.6), as were rate of CSOM 9% and 8%, respectively (RD −1% [95% CI −5 to 2] p = 0.4). Of 401 PCV7 children and 424 PHiD-CV10 children with bilateral otoscopic assessments, 34% and 45%, respectively had bilateral OME; 6% in each group had unilateral OME (Table 5).

**Table 5:** Prevalence of all forms of otitis media including combination categories, by vaccination groups

	**PCV7 444**		**PHiD-CV10 451**		**Absolute risk difference [95%CI]**	**P value**
	**n**	**%**	**n**	**%**		
No. children without assessment	12	3%	14	3%		
No. children with diagnosis based on tympanometry alone	4	1%	0	0%		
No. children with unilateral otoscopy	27	6%	13	3%		
No. children with bilateral otoscopy	401	90%	424	94%		
No. children with at least one ear successfully assessed	432	97%	437	97%		
**Clinical diagnosis**	**PCV7 432**		**PHiD-CV10 437**			
normal	32	7%	41	9%	2% [−1 to 6]	0.29
OME	175	41%	222	51%	10% [3-17]	**0.002**
AOMwoP	150	35%	111	25%	−9% [−15 to −3]	**0.003**
AOMwiP	27	6%	24	5%	−1% [−4 to 2]	0.63
Dry Perforation	8	2%	5	1%	−0.7% [−2 to 1]	0.39
CSOM	40	9%	34	8%	−1% [−5 to 2]	0.43
Bilateral OME*	136/401	34%	191/424	45%	11% [4-17]	**0.001**
Unilateral OME*	24/401	6%	25/424	6%	0.1% [−3 to 3]	**1.000**
**Combination OM categories**						
Any suppurative OM (AOMwoP, AOMwiP, CSOM)	217	51%	169	39%	−12% [−19 to −5]	**0.0004**
Any TMP (AOMwiP, DP, CSOM)	75	17%	63	14%	−3% [−8 to 2]	0.24
Any ear pain (today or last night)	34/408	8%	17/337	5%	−3% [−7 to 0.2]	0.08
**Non-mixed PCV schedules**	PCV7-only 353**		**PHiD-CV10-only 319**			
No. children with at least one ear successfully assessed	345	98%	309	97%		
Normal	24	7%	31	10%	3% [−1 to 7]	0.16
OME	127	37%	152	49%	12% [5 to 20]	**0.001**
Any suppurative OM (AOMwoP, AOMwiP, CSOM)	191	56%	121	40%	−16% [−24 to −8]	**<0.0001**
Any TMP (AOMwiP, DP, CSOM)	62	18%	48	16%	−2% [−8 to 3]	0.40

*Laterality of OME could only be determined where bilateral assessments were successful; 401 PCV7 children and 424 PHiD-CV10 children had bilateral assessments.

**138 children in the PCV7-only group had also received PPV23.

For combination diagnostic categories, the PHiD-CV10 group had significantly less suppurative OM (39% versus 51%, RD −12% [95% CI −19 to −5] p = 0.0004) and similar rates of TMP (14% versus 17%. RD −3% [95% CI −8 to 2] p = 0.2) compared to the PCV7 group. Very few mothers (~6%) reported that their child had had ear pain on the day or during the night prior to the ear assessment (Table 5).

Comparisons of OM prevalence between recipients of non-mixed PCV schedules (353 PCV7- and 319 PHiD-CV10-recipients) showed almost identical differences in prevalence of OME (RD = 12% [95% CI 5 to 20] p = 0.001), any suppurative OM (RD = −16% [95% CI −24 to −8] p < 0.0001), and any TMP (RD = −3% [95% CI −8 to 3] p = 0.4) as were seen for the primary comparisons of recipients of at least 2 doses of PCV and not more than one dose of an alternative PCV (Risk Differences of 10%, −12% and −3% for OME, any suppurative OM and any TMP, respectively) (Table 5).

PPV23 was received by 138 children in the PCV7 group. In a post-hoc analysis, we found that compared to 39 age-eligible children in the PCV7 group who did not receive PPV23, there was no significant association between PPV23 and suppurative OM, although TMP was significantly more common in the PPV23 group (24%) than the non-PPV23 vaccinated group (8%) (RD 16%, 95% CI 5 to 27] p = 0.03, data not shown).

#### Age and any suppurative OM

A consistent difference in vaccine group prevalence of suppurative OM was seen in all age groups up to 24 months of age (Table 6).

**Table 6:** Prevalence of any suppurative OM in each age group, by vaccination group

	PCV7 424*		PHiD-CV10 432*		**Absolute risk difference [95%CI]**	**P value**
**Age group**	**n**	**%**	**n**	**%**		
3 to <6	4/8	50%	3/9	33%	−17% [−63 to 30]	0.48
6 to <9	34/47	71%	33/60	55%	−16% [−34 to 2]	0.09
9 to <12	27/38	71%	21/45	47%	−24% [−45 to −4]	**0.03**
12 to <18	50/84	59%	46/114	40%	−19% [−33 to −5]	**0.008**
18 to <24	54/98	55%	39/101	39%	−16% [−30 to −3]	**0.02**
24 to <36	48/148	32%	27/103	26%	−6% [−18 to 5]	0.29

*Denominator: Dry perforation (8 children in the PCV7 group and 5 children in the PHiD-CV10 group) have been excluded.

Any suppurative OM any AOMwoP (Acute Otitis Media without Perforation), any AOMwiP (Acute Otitis Media with Perforation), or any CSOM (Chronic Suppurative Otitis Media).

*CI*, Confidence Interval.

*PCV7*, 7-valent pneumococcal conjugate vaccine.

*PHiD-CV10*, 10-valent pneumococcal *Haemophilus influenzae* protein D conjugate vaccine.

### Univariate and multivariate analyses of risk factors for any suppurative OM

In a univariate analysis adjusting for community, PHiD-CV10 compared to PCV7 was significantly negatively associated with any suppurative OM (Odds Ratio = 0.65 [95% CI 0.5 to 0.9] p = 0.003) (Table 7). Univariate analyses of other risk factors for any suppurative OM showed increased risk as the additional number of children under the age of 5 years per household increased (OR 2.4 [95% CI 1.5 to 4] p = 0.001 for the third child) and reduced risk as age of the child increased (OR 0.33 [95% CI 0.2 to 0.5] p < 0.0001, for the third year). Females were at increased risk compared to males (OR 1.4 [95% CI 1.1 to 1.9] p = 0.02). Antibiotic prescribing within 5 weeks of assessment had no significant impact on prevalence of any suppurative OM (OR 1.2 [95% CI 0.9 to 1.6] p = 0.2). There was also no significant association of suppurative OM with abnormal haemoglobin, any child care attendance, having a sibling with perforation history, maternal smoking, being near a camp fire, maternal age or education, breast feeding, pacifier use, or gestational age less than 32 weeks (Table 7).

**Table 7:** Univariate and multivariate analyses of risk factors for suppurative OM, adjusted for community

	With suppurative OM**	**UNI-VARIATE OR**	**95% CI**	**p**	**MULTI-VARIATE OR**	**95% CI**	P*
Vaccine group							
PCV7	51%						
PHiD-CV10	39%	0.64	0.48 to 0.86	**0.003**	0.58	0.41 to 0.80	**0.001**
Children under age of 5 years in household							
0	39%			**0.003**			**0.009**
1	47%	1.43	0.98 to 2.10		1.34	0.91 to 1.98	
2	53%	1.85	1.12 to 3.07		1.66	0.99 to 2.80	
3+	60%	2.42	1.46 to 3.99		2.36	1.41 to 4.00	
Age group (years)							
< 1 year	58%			**0.000**			**0.000**
1 to < 2 years	47%	0.70	0.49 to 1.0		0.72	0.50 to 1.04	
2 to < 3 years	30%	0.33	0.22 to 0.49		0.31	0.20 to 0.47	
Gender							
Male	41%						
Female	49%	1.41	1.07 to 1.86	**0.016**	1.46	1.07 to 1.92	**0.016**
Antibiotics prescribed in previous 5 weeks							
None	44%						
Any	47%	1.21	0.90 to 1.62	0.20	1.24	0.91 to 1.69	0.17
Haemoglobin							
> = 11 mg/dL	42%						
<11 mg/dL	49%	1.28	0.93 to 1.75	0.13			
Child care attendance							
none	47%						
any	47%	1.03	0.64 to 1.67	0.91			
Sibling history of OM “runny ears’							
No	47%						
Yes	47%	0.98	0.69 to 1.39	0.90			
Maternal smoking							
No	46%						
Yes	48%	1.11	0.80 to 1.53	0.53			
Child near campfire last week							
No	48%						
Yes	47%	0.89	0.63 to 1.35	0.51			
Maternal age at birth of this child							
> = 21 yrs	43%						
< 21 yrs	52%	1.39	0.97 to 1.99	0.07			
Maternal education							
no certificate	46%						
certificate	49%	1.07	0.74 to 1.57	0.71			
Breastfed							
Never	51%						
Some	47%	0.86	0.45 to 1.65	0.65			
Pacifier							
Never	47%						
Some	47%	1.02	0.70 to 1.54	0.91			
Gestational age							
> = 32 weeks	45%						
< 32 weeks	54%	1.3	0.60 to 3.06	0.51			

*Bold signifies P value less than 0.05.

**Analysis of 383 children with and 465 children without suppurative OM.

A random-effects logistic regression model (with adjustment for inconsistent community representation) was conducted with complete data from 465 children without suppurative OM and 383 with suppurative OM. We found that compared to vaccination with PCV7, PHiD-CV10 vaccination was associated with less suppurative OM (Odds Ratio, OR = 0.6 [95% CI 0.4 to 0.8] p = 0.001) as was age (OR 0.3 [95% CI 0.2 to 0.5] p < 0.0001 for the third year). Living with more than 3 other children under 5 years of age (OR = 2.4 [95% CI 1.4 to 4.0] p = 0.001) and female gender (OR 1.5 [95% CI 1.1 to 1.9] p = 0.02) were also associated with increased suppurative OM. Recent antibiotic prescribing was not associated with increased or decreased rates of suppurative OM (Table 7).

## Discussion

Our surveillance highlights the poor general health, particularly poor ear health, of Australian Indigenous children living in remote communities during the PCV era. Although the children we assessed were generally not considered unwell by their careers and were not presenting for ear health services, nine of every ten children in each vaccine group had some form of OM. Most of these children had forms of OM that required medical and/or audiological referral and possibly otolaryngologist review. In addition, one in four or five children had skin problems, more than one third had runny nose and just under one third had a cough. Antibiotics had been recently prescribed for more than one in four children. Although few children under the age of 6 months were seen, the early age of OM onset remains a huge challenge and requires new strategies for prevention.

Our comparison found a 12% absolute reduction in any suppurative OM (either AOMwoP, AOMwiP or CSOM) including a small non-significant 3% reduction in any TMP (AOMwip, DP or CSOM), and a concomitant 10% increase in OME, in the PHiD-CV10 group compared to the PCV7 group. Importantly the difference in any suppurative OM was present from an early age, reaching statistical significance after 9 months of age.

Implications of this shift from suppurative to non-suppurative disease could reduce rates of antibiotic prescribing and change demand for surgical procedures such as tympanoplasty and tympanostomy tubes.

These improvements in ear health occurred whilst prevalence of skin, cough and runny nose increased, suggesting that there was no strong temporal trend toward improved child health that might explain the improvement in ear health.

Sampling was reliant on community approval which varied each year of surveillance. We previously identified considerable community variation in prevalence of CSOM [1]. In an analysis restricted to the data from those communities that were sampled in both eras (data not show), there were similar differences in AOMwoP and any suppurative OM, and a significant increase in OME for the PHiD-CV10 group compared to the PCV7 group. Furthermore, the adjustment for inconsistent community representation (using xtset in Stata) did not change the independent association between vaccine and severity of OM.

We did not use random selection of participants, but aimed to assess all eligible children in the community. It is possible that this would bias selection for children with healthy ears if families of children with poor ear health refused participation, or bias towards selection of unhealthy ears if families thought the assessment might be beneficial. Whether such bias existed in our study, or whether bias, if it existed, shifted between the two vaccine era periods was not determined.

Eighty nine children in the PCV7 group had also received a single dose of PHiD-CV10, and 40 children in the PHiD-CV10 group had also received a single dose of PCV7. Ninety children in the PHiD-CV10 group and 2 in the PCV7 group also received a single dose of PCV13. Comparisons of OM prevalence between recipients of non-mixed PCV schedules showed very similar differences in prevalence between PCV groups, which supports a hypothesis that an additional single dose of an alternative PCV vaccine may have no detectable impact on clinical outcome. We have not presented a comprehensive separate analysis of the impact of a booster PPV23 dose. There was no significant association between PPV23 and suppurative OM although significantly more PPV23-vaccinated children had TMP (data not shown).

Our comparison of risk factors was potentially compromised by the large number of carers in the PHiD-CV10 group who refused consent for the risk factor questionnaire. Nonetheless, the ear health of PHiD-CV10 children for whom we did not get consent for the questionnaire was similar to that of PHiD-CV10 children for whom we did have consent, so we consider the consent refusal to be unrelated to ear health.

Household crowding [12] and child care attendance [13,14] are known to be associated with increased risk of OM. The PHiD-CV10 group had a lower mean household number of children under the age of 5 years compared to the PCV7 group and significantly greater exposure to recent beta-lactam antibiotics. The antibiotics prescribed could be either protective [15-17], or indicative of a recent OM episode. The multivariate analysis of risk factors for suppurative OM confirmed that recent antibiotic prescribing was neither a significant risk nor protective factor for suppurative OM. Thus, three key factors have been found to be associated with the reduction in suppurative OM and concomitant increase in OME; i) PHiD-CV10 was protective compared to PCV7, ii) living in a household with more children less than 5 years of age per household was a significant risk factor, and iii) older age is protective of suppurative OM. Recent prescribing of antibiotics, child care attendance and other measured factors were not associated with suppurative OM.

Our data must be interpreted with caution since most (70%) children vaccinated with PCV7 were seen between 2009 and 2010 whereas most (87%) PHiD-CV10 children were seen between 2011 and 2012. Factors not measured may also have changed during this period. In addition, PHiD-CV10 has recently been shown to have no impact on nasopharyngeal (NP) carriage of non-typeable *H. influenzae*[18]. Whilst we understand the NP to be the source of pathogens that cause middle ear infections, it is feasible that vaccine induced immune responses can deliver protection in the middle ear without reducing NP carriage. This hypothesis is plausible (not all NP colonised children have OM), and should be confirmed in randomised controlled vaccine trials with OM outcomes and concomitant studies of NP microbiology.

## Conclusion

Our study identifies ongoing high rates of ear disease and other infections in Indigenous children living in remote regions. A comparison of ear health of children receiving PCV7 or PHiD-CV10 according to the NT childhood vaccination schedules shows that PHiD-CV10 was associated with a 12% reduction in suppurative ear disease (AOM without perforation, AOM with perforation and CSOM combined) and a concomitant increase in OME. Multivariate logistic regression did not change these findings. Clinical trials of vaccines that offer broader coverage of OM pathogens and that are effective from an earlier age are urgently needed in this highly disadvantaged population.

## Abbreviations

AOMwiP: Acute otitis media with perforation; AOMwo: Acute otitis media without perforation; CI: Confidence Interval; CSOM: Chronic suppurative otitis media; DP: Dry perforation; IPD: Invasive pneumococcal disease; Mcat: Moraxella catarrhalis; Mo: Month; NT: Northern Territory; NTHi: Non-typeable *Haemophilus influenzae*; OM: Otitis media; OME: Otitis media with effusion; PCV7: 7-valent Pneumococcal conjugate vaccine; PCV13: 13-valent Pneumococcal conjugate vaccine; PHiD-CV10: 10-valent Pneumococcal *Haemophilus influenzae* protein D conjugate vaccine; PPV23: 23-valent pneumococcal polysaccharide vaccine; RD: Risk difference; SD: Standard deviation; TMP: Tympanic membrane perforation.

## Competing interests

Name: Amanda Leach. Prof Amanda Leach has received research funding from GlaxoSmithKline and Pfizer. Name: A/Prof Ross Andrews has received research funding from GlaxoSmithKline. Name: Heidi Smith-Vaughan. Dr Heidi Smith-Vaughan has received research funding from GlaxoSmithKline. Name: Peter Morris. A/Prof Peter Morris has received research funding from GlaxoSmithKline. No other authors have any conflicts of interest to disclose.

## Authors’ contributions

AJL conceptualised and designed the study, obtained funding, obtained ethical approvals, oversaw the project, drafted the initial manuscript, conducted the analyses. CW managed the project, collected majority of data at most sites, reviewed the manuscript. RA conceptualised and designed the study, obtained funding, critically reviewed draft manuscript. MC conducted the analyses critically reviewed draft manuscript. HSV critically reviewed draft manuscript. PSM conceptualised and designed the study, obtained funding, obtained ethical approvals, oversaw the project, critically reviewed draft manuscript. All authors read and approved the final manuscript as submitted.

## Pre-publication history

The pre-publication history for this paper can be accessed here:

http://www.biomedcentral.com/1471-2431/14/200/prepub
